# Poor Glycemic Control Affecting Screening of Prostate Carcinoma

**DOI:** 10.7759/cureus.58680

**Published:** 2024-04-21

**Authors:** Archana Bharti, Ravi Shekhar, Pritam Prakash, Sweta Kumari, Santosh Kumar

**Affiliations:** 1 Biochemistry, Indira Gandhi Institute of Medical Sciences, Patna, IND; 2 Biochemistry, Indra Gandhi Institute of Medical Sciences, Patna, IND

**Keywords:** metformin, diabetes mellitus, carcinoma prostrate, prostrate specific antigen, glycated hemoglobin

## Abstract

Introduction

Diabetes and cancer are commonly linked together. The possible links include insulin resistance, hyperinsulinemia, hyperglycemia, oxidative stress, and chronic inflammation. These are factors that have potential promoting effects on the progression of cancer in many ways. Measurement of prostate-specific antigen (PSA) is widely applied for early detection of prostate cancer. However, several factors influence serum PSA levels in men including age, benign prostatic hyperplasia, prostatitis, and body mass index (BMI). The risk of several malignancies is increased in diabetes but the risk of prostate carcinoma in diabetic patients is reduced secondary to lowering of testosterone levels during the state of hyperinsulinemia. A negative association between serum PSA levels and metformin use is also an explanation of low cancer prostate incidence with diabetes.

Objective

The study aims to evaluate the PSA levels in diabetic patients with poor glycemic control i.e., glycated hemoglobin (HbA1c) ≥ 7%) vs good glycemic control (HbA1c < 7%).

Materials and methods

Samples of PSA in diabetic patients collected in the Department of Biochemistry at Indira Gandhi Institute of Medical Sciences (IGIMS), Patna, were included. The observational study was carried out on clinically diagnosed 318 cases of diabetes attending both the outpatient and inpatient Department, IGIMS, Patna. Six ml venous blood samples were collected from patients after obtaining informed consent and ethical clearance. Patient details regarding age, complete clinical details, and general physical examinations were recorded. Serum levels of PSA, fasting plasma glucose (FPG) and HbA1c were analyzed and values were compared.

The serum level of PSA was estimated by the chemiluminescent immunoassay (CLIA) method on an automated immunoassay analyzer in the Department of Biochemistry, maintaining all the quality control precautions using a control, calibrator, and reagent kit. HbA1c estimation was by chromatography technique. Fasting plasma glucose was estimated using the hexokinase method on an automated chemistry analyzer. Statistical analyses were performed using SPSS software, version 16.0 (SPSS Inc., Chicago). The median and interquartile range were calculated for numerical variables. Covariance analysis was used in the comparisons among groups. The Mann-Whitney U test was applied to detect the comparison between the two groups. Significance was determined by the P value. P value<0.05 was considered significant.

Result

Serum PSA value was found to be higher in (the good glycemic control group) with a median of 0.99 with an interquartile range of 3.14, than in (the bad glycemic control group) with a median of 0.49 with an interquartile range of 3.9, and the difference is statistically significant. The difference is also statistically significant in the subgroup (i) with PSA value <4 ng/ml. In subgroups (ii) and (iii), PSA values 4 ng/ml-8 ng/ml and PSA values >8 ng/ml respectively, no significant differences were found.

Conclusion

It was found that serum prostate-specific antigen levels have been lower in diabetic patients with poor glycemic control than in good glycemic control. Future studies with a larger sample size and detailed information on diabetes duration and management are recommended.

## Introduction

Diabetes and cancer are common diseases worldwide, with a rising global incidence and prevalence and a tremendous impact on health. The two most common chronic diseases affecting the ageing male population are type 2 diabetes mellitus (T2DM) and prostate cancer. The prevalence of diabetes in India is 11.4%, as reported by the Indian Council of Medical Research-India Diabetes (ICMR INDIAB) study published in 2023 [1].

Approximately 90-95% of individuals with diabetes have T2DM, and the risk increases with age, obesity, and a sedentary lifestyle. T2DM is a complex metabolic disease characterized by insulin resistance and hyper-insulinemia [2]. Several studies have suggested that T2DM significantly increases the risk for different cancers but is associated with a decreased risk of prostate cancer. Cancer and T2DM share many risk factors, but the link between the pathogenesis of the two diseases is still incompletely understood [3].

Prostate cancer is the sixth most common cancer in India, as per the 2018 GLOBOCAN data, with an age-adjusted incidence rate of 10.2/100,000 per year and age-adjusted mortality rate of 4.2/100,000 population [4]. Serum prostate-specific antigen (PSA) level is found to be higher (>10 ng/ml) in Indian men at the time of diagnosis of prostate cancer than in their Western counterparts. The incidence of metastasis is also higher in Indian men than in the Western population. [4] Early prostate cancer is usually asymptomatic, and an elevated serum PSA level is the first screening test for malignancy. However, serum PSA is still a controversial screening test for healthy, asymptomatic males, as elevated serum PSA is found in many benign prostatic conditions such as infection, trauma, inflammation, and benign prostatic hyperplasia [5].

Prostate-specific antigen (PSA) is a serine protease in semen produced by the columnar epithelium of prostatic tissue. Cancer cells do not make more PSA than normal cells, but malignant cells, lacking a basal layer, will more easily release PSA into the surrounding extracellular fluid, eventually raising blood levels. It is a very sensitive screening test but is relatively non-specific as elevated values of serum PSA are found both in benign and malignant diseases of the prostate. According to the American Urology Association and the European Association of Urology, serum PSA is the first screening test for prostate carcinoma [5]. Serum PSA, in combination with age, prostate volume, and prostate cancer risk calculators, enables improved screening of men with prostate cancer. However, many factors affect serum PSA levels, such as age, race, medications, acute prostatitis, benign prostate hyperplasia, body mass index (BMI), and diabetes.

In the case of race, in black men,40% of cancers would be missed with the use of traditional cut-off values of PSA. In men aged >50 years, the possibility of prostate cancer in patients with serum PSA levels of 2.5-4 ng/mL, >4 ng/mL, and >10 ng/mL was 27.0%, 20-30%, and 42-64%, respectively [6]. Obesity or high BMI tends to lower PSA values because of the larger intravascular volume and dilution effect [7]. The patient’s general health is also an essential factor in the decision to screen for prostate cancer, as guidelines recommend against screening individuals with a life expectancy of fewer than 10 years.

An article on “Prostate Cancer Screening” recommended that annual PSA screenings for prostate cancer must be started at the age of 45 years in high-risk individuals (a family history of either prostate cancer or multiple cancers, known high-risk germline mutations such as *BRCA 2* or African ethnicity) and at 50 years of age for men at average risk, after informing them about the pros and cons of the screening test. It recommended that testing be continued until the age of 70-75 years [8].

Cancer societies recommend different cut-off values of serum PSA for prostate cancer screening. According to the American Cancer Society and the American Urological Society, most men without prostate cancer have PSA levels below 4 ng/mL of blood. With a PSA value greater than 4 ng/mL, the specificity of detection of prostate cancer approaches 91%. Values above 4 ng/ml and less than 8 ng/ml are in the borderline range. For higher PSA levels, diagnostic tests such as a prostate biopsy are recommended. A serum PSA of > 10 ng/ml increases the chance of having prostate cancer by over 50%.PSA levels are higher in older age groups than in younger men, even when there is no cancer [9,10]. The Indian Urology Society recommends personalized risk stratification after 50 years when the life expectancy is >10-15 years, and at-risk men must undergo serum PSA and digital rectal examination. No recommendation has been made on the cut-off of PSA values for a prostate biopsy. However, a PSA value of 4 ng/ml has been considered as a cut-off value for further evaluation [4]. Serum PSA levels have also been associated with the severity of the disease. Serum PSA values >20 ng/mL have a positive predictive value of 65% for metastatic disease and skeletal involvement and 86% if PSA levels are >100 ng/ml. Serum PSA values <10 ng/mL are rarely found with metastatic disease. So, bone scans in prostate cancer are not generally recommended unless the PSA level is >20 ng/ml [3].

Serum PSA has an inverse association with T2DM. An inverse association of diabetes with the incidence of prostatic cancer has also been noted. This inverse association is mainly seen with non-aggressive cancer, suggesting that diabetes may mask the diagnosis by influencing the frequency and interpretation of screening tests (PSA). In contrast, higher insulin levels have been associated with a higher rate of prostate cancer [11]. Insulin is a member of the growth factor family that includes insulin-like growth factor-I (IGF-I) and IGF-II and has important mitogenic effects. Insulin resistance, hyperglycemia, dysregulation of sex hormones, oxidative stress, and inflammatory cytokines play a significant role in cancer development [12]. Reduced testosterone levels in diabetes may explain the reduced incidence of prostate cancer in diabetes, as testosterone promotes prostate cell growth. However, the use of statins and metformin and changes in diet and lifestyle for the control of diabetes affect the serum PSA that is used as a screening tool for prostate cancer, masking the diagnosis. Low PSA levels among males with diabetes might lead to a detection bias and show the inverse association [13].

Many studies have been published stating the inverse association between T2DM and PSA. However, the effect of the severity of T2DM and poor glycemic control on serum PSA concentration has rarely been studied. According to the American Diabetes Association 2024, assessment of glycemic status is recommended with glycosylated hemoglobin (HbA1c) or continuous glucose monitoring metrics at least twice a year. More frequent assessment is required for individuals not meeting treatment goals. For adults, an HbA1c goal of <7% (53 mmol/mol) without significant hypoglycemia is appropriate [14,15]. The goal is to keep the microvascular complications of diabetes in check. Among people with T2DM, the Kumamoto Study [2] and the UK Prospective Diabetes Study (UKPDS) [16] examined the effects of “intensive glycemic control.”Mean HbA1c was 7.1% vs 9.4% in the Kumamoto Study and 7.0% vs 7.9% in (UKPDS). They confirmed the increased microvascular complications in the groups, with HbA1c of 7.9% and 9.4%, than the group with HbA1c of up to 7.1% and 7.0%, respectively. Hence, we decided to study the effect of poor glycemic control on serum PSA levels. Poor glycemic control has been assessed based on an HbA1c ≥ 7%.

## Materials and methods

This observational study was performed in the Department of Biochemistry, Indira Gandhi Institute of Medical Sciences (IGIMS), Patna, India. The study protocol was approved by the Institutional Ethics Committee (no. 867/IEC/IGIMS/2023). Serum samples of 1439 patients were analyzed for PSA in the clinical biochemistry laboratory between March 2023 and July 2023. After the informed consent form was received, a detailed medical history was obtained from the patients. There were 380 blood samples from diabetic patients with confirmed diagnoses. Three hundred and eighteen patients with a history of diabetes who were screened for carcinoma prostate with serum PSA level were enrolled. Exclusion criteria were (i) diagnosed case of prostate cancer or any other cancer, (ii) those taking α-blockers, phosphodiesterase inhibitors, or 5α-reductase inhibitors, (iii) men with creatinine levels > 5 mg/dl, hepatic dysfunction (high serum transaminase levels), (iv) leading sedentary lifestyle. Thus, 62 diabetic patients were excluded.

PSA, fasting plasma glucose, and HbA1c measurements

PSA, fasting plasma glucose, and HbA1c were measured in venous blood samples. Venous blood 2 ml was collected in a serum separator vial for PSA, an ethylenediamine tetraacetic acid (EDTA) vial for HbA1C, and a sodium fluoride-containing vial for plasma glucose estimation. All the tests were performed after a third-party internal quality control check. Three levels of quality control were checked before testing PSA, and two levels were run for fasting plasma glucose and HbA1C tests. Serum PSA analyses were done using a fully automated chemiluminescence-based immunoassay analyzer. Fasting plasma glucose was analyzed using the hexokinase method on an automated chemistry analyzer. HbA1c analysis was done using a high-performance liquid chromatography technique-based auto-analyzer on EDTA samples. All measurements were done in the clinical biochemistry laboratory, IGIMS, Patna, according to the manufacturer’s instructions.

Statistical analyses

All statistical analyses were completed using SPSS software, version 16.0 (SPSS Inc., Chicago). Mean, standard deviation, median, minimum, and maximum values were used for descriptive data. Correlations between serum HbA1c levels and PSA were examined using the Spearman rank test. The normality of data was checked by the Kolmogorov-Smirnov test. The continuous variables, presented as medians, were tested using the Mann-Whitney U test. For all statistical comparisons, significance was considered as a p < 0.05.

## Results

A total of 1,439 samples for PSA screening were considered. Of these, 318 were found to be those of diabetic men. Groups were formed based on the HbA1c values: Group A consisted of patients with an HbA1c <7%, and Group B patients with an HbA1c >7%. In both groups, three subgroups were defined based on serum PSA concentration. (i) PSA 0-4 ng/ml, (ii) PSA 4-8 ng/ml and (iii) PSA>8 ng/ml. The PSA values in Group A and Group B were compared. There were 159 samples in Group A (i), 16 in A(ii), and 31 in A(iii). There were 83 samples in Group B (i), 10 in B (ii), and 19 in B (iii).

Spearman rank correlation coefficient was calculated. Correlation coefficient of PSA and HbA1c in group A was 0.025 with p value of 0.725, and in group B -0.232 with p value of 0.094. In different subgroups of group A and then in group B (Tables 1, 2). In Group A, subgroups (i) and (ii) i.e., serum PSA level of <4 ng/ml and 4-8 ng/ml, the correlation coefficient was -0.107 and -0.062, respectively. In subgroup (iii) i.e., serum PSA level of >8 ng/ml correlation coefficient was 0.143. In Group B, subgroup (i) i.e., serum PSA level of <4 ng/ml correlation coefficient was 0.305. In subgroups (ii) and (iii) i.e., serum PSA levels of 4-8 ng/ml and >8 ng/ml correlation coefficient was -0.122 and -0.023, respectively.

**Table 1:** Spearman rank correlation between PSA and different diabetes parameters in Group A (HbA1c<7%) HbA1c: Glycated hemoglobin, PSA: Prostate-specific antigen, FBS: Fasting blood glucose

	ρ	P value
HbA1C<7		
(PSA < 4)		
PSA Vs HbA1C	-0.107	0.18
PSA Vs FBS	-0.107	0.18
(PSA 4 TO 8)		
PSA Vs HbA1C	-0.062	0.82
PSA Vs FBS	-0.056	0.837
(PSA > 8)		
PSA Vs HbA1C	0.143	0.443
PSA Vs FBS	0.128	0.492

**Table 2:** Spearman rank correlation between PSA and different diabetes parameters in Group B (HbA1c >7%) HbA1c: Glycated hemoglobin, PSA: Prostate-specific antigen, FBS: Fasting blood glucose

HbA1C ≥7		
(PSA < 4) ρ P value		
PSA Vs HbA1C	0.305	0.005
PSA Vs FBS	0.188	0.089
(PSA 4 TO 8)		
PSA Vs HbA1C	-0.122	0.738
PSA Vs FBS	-0.122	0.738
(PSA > 8)		
PSA Vs HbA1C	-0.023	0.926
PSA Vs FBS	-0.028	0.909

**Figure 1:**
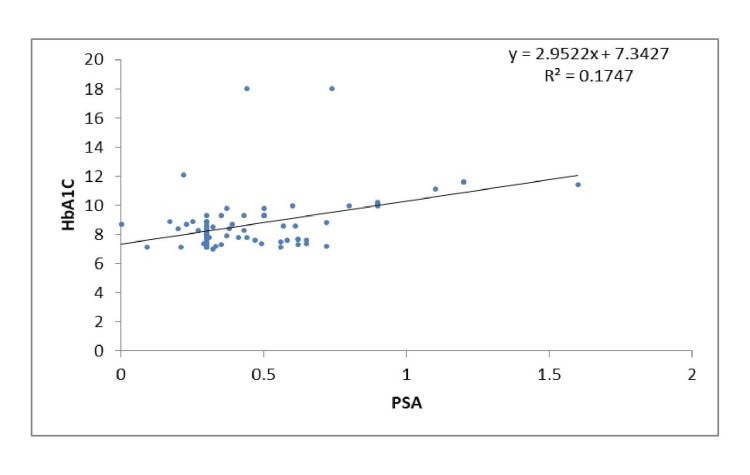
Scatter plot showing a correlation between HbA1c and PSA in subgroup B (i) HbA1c: Glycated hemoglobin, PSA: Prostate-specific antigen

There was an inverse association between PSA and fasting blood glucose and HbA1c in groups A (i) and (ii), but in group A(iii), the association was positive; however, it was not statistically significant. In group B, a significant positive association was found in the subgroup (i) (Figure 1), and a nonsignificant negative association was found in subgroups (ii) and (iii).

Mann Whitney U test found a statistically significant difference in Groups A and B in subgroup (i). No statistically significant difference was found between Groups A and B in subgroups ii and iii. Overall, the serum PSA value was found to be higher in Group A (good glycemic control group) with a median of 0.99 with an interquartile range of 3.14 compared with Group B (poor glycemic control group), where the median was 0.49 with an interquartile range of 3.9. The difference was statistically significant (Table 3).

**Table 3:** Statistical analysis results Comparison of median prostate-specific antigen (PSA) levels (interquartile range) in groups A (good glycemic control) and B (poor glycemic control); Mann Whitney U Test, Median (Inter Quartile Range).

	n	Group A HbA1C <7%	Group B HbA1C ≥7%	p-value*
PSA	319	0.99 (3.14)	0.495 (3.9)	<0.05
PSA < 4 ng/ml	242	0.710 (0.9)	0.32 (0.3)	< 0.05
PSA 4–8 ng/ml	26	6.82 (2.8)	5.1 (2.5)	0.187
PSA > 8 ng/ml	50	21.3 (32.9)	25 (65.6)	0.358

## Discussion

The relationship between diabetes mellitus (DM) and prostate cancer has been frequently studied in the last 10 years. Evidence supports an inverse association between the two conditions. Hypotheses have been given to explain this inverse association. Reduction of testosterone levels in long-term diabetes or diabetes-induced microvascular damage in the prostate has been postulated to have a protective effect [17]. On the other hand, the inverse relationship may be due to the masking of the diagnosis of prostate cancer among people with diabetes due to lower PSA levels. People with diabetes and a low PSA would be less likely to be subjected to a biopsy and, as a result, be diagnosed late and with a higher grade of disease as compared with people with no diabetes [18].

Several studies have reported an inverse relation of PSA levels with T2DM. Werny et al. [19] found that men with diabetes had a 21.6% lower mean PSA level than those without diabetes in age-matched samples. Fukui et al. [20] found that serum PSA levels were 10-16% lower in diabetic men than in healthy Japanese men, excluding the age group of 40-49 years. Bernal-Soriano et al. [21] also concluded in a meta-analysis that diabetic men showed lower PSA levels than non-diabetics.

We observed an overall lower value of serum PSA in the poor diabetic control group than in the good diabetic control group. However, this relation was statistically significant only if the PSA was 0-4 ng/ml, suggesting masking of the diagnosis may be found in this subgroup. A clear negative correlation between PSA and HbA1c was not found, so this can be due to the effect of anti-diabetic drugs, as metformin and insulin are often used as first-line hypoglycemic drugs. Metformin has been related to decreasing prostate cancer, while Insulin is found to increase prostate cancer [13].

Atalay et al. [13] suggested a significant impact of poor glycemic control on serum PSA levels in T2DM. The possible explanations given by them were obesity, medications for dyslipidemia, microvascular complications (which may include prostate ischemia), and lower serum androgen levels in T2DM. Sarma et al. also found similar results, concluding that men with an HbA1c of 6.1-6.9% and ≥7% had 15% (p =0.004) and 29% (p =0.003) lower serum PSA compared with men with a normal HbA1c [22].

However, diabetes is associated with an increased cancer risk, explained by insulin-like growth factor 1 (IGF-1), IGF-1R, and the signaling pathway. Many studies have proven an increased risk of prostate cancer and high-grade tumors in diabetic patients. The role of anti-diabetic drugs has also been found to be significant in PSA and prostate cancer. Murtola et al. [11], in a population-based cohort study, have concluded that elevated diabetic fasting blood glucose level is associated with elevated prostate cancer risk, especially in the context of systematic PSA-based screening. However, the use of anti-diabetic drugs removed the risk association, supporting a risk-lowering effect or masking effect of these drugs. In a meta-analysis, Liu et al. [23] concluded that serum IGF-1, IGF-1R, and its signaling pathway are similar to insulin signaling and have been recognized to play an essential role in the development of prostate cancer. Targeting IGF-1R has also been researched as a novel cancer therapy. According to Cohen et al. [24], as PSA is a serine protease found in semen, cleavage of insulin-like growth factor-binding protein 3 (IGFBP-3) by PSA resulted in a marked reduction in the binding affinity of the fragments to IGF-I but not IGF-II. So, they concluded that PSA may have a role in prostate cancer by altering IGF-IGFBP-3 interactions.

Prostate epithelial cell growth is also seen downregulated by metformin, which may result in lower PSA levels, masking the presence of prostate cancer or delaying its detection, leading to an apparent reduced risk. Sulfonylureas and insulin analogs, which are commonly used as second- and third-line therapies for diabetes, may lead to increased insulin-like growth factor levels and hence promote prostate cancer growth and simultaneously increase PSA levels. Beckmann et al. [25], Atale et al. [13], and Feng et al. [26] concluded that the presence of diabetes is inversely associated with prostate cancer risk, particularly in localized and low and intermediate-grade prostate cancer [26]. For advanced cancer, reduced risks were also observed but were not statistically significant. These findings are similar to our findings in subgroup ii. 

The limitation of our study is that the sample size was small, so the study’s power was low. Another potential limitation is the lack of data regarding diabetes duration, treatment duration, and level of adherence and control, which might also affect PSA values. Hence, further longitudinal studies are needed to identify whether there is a masking effect of persistent hyperglycemia on cancer prostate diagnosis.

## Conclusions

The study aimed to evaluate the PSA levels in diabetic patients with poor glycemic control i.e., glycated hemoglobin (HbA1c) ≥ 7%) vs good glycemic control (HbA1c < 7%). It was found that serum PSA levels were low in diabetic patients with poor glycemic control as compared with those with good glycemic control. Thus, either treatment of diabetes or lifestyle changes may decrease PSA levels. Or perhaps prostate cancer incidence decreases in poor glycemic control conditions. Future studies with a larger sample size and detailed information on diabetes duration and management are needed.
